# Analysis of five-year trends in self-reported language preference and issues of item non-response among Hispanic persons in a large cross-sectional health survey: implications for the measurement of an ethnic minority population

**DOI:** 10.1186/1478-7954-8-7

**Published:** 2010-04-22

**Authors:** William S Pearson, William S Garvin, Earl S Ford, Lina S Balluz

**Affiliations:** 1 Division of Adult and Community Health, Centers for Disease Control and Prevention, Atlanta, GA, USA; 2 Office of Surveillance, Epidemiology and Laboratory Services, Centers for Disease Control and Prevention, Atlanta, GA, USA

## Abstract

**Background:**

Significant differences in health outcomes have been documented among Hispanic persons, the fastest-growing demographic segment of the United States. The objective of this study was to examine trends in population growth and the collection of health data among Hispanic persons, including issues of language preference and survey completion using a national health survey to highlight issues of measurement of an increasingly important demographic segment of the United States.

**Design:**

Data from the 2003-2007 United States Census and the Behavioral Risk Factor Surveillance System were used to compare trends in population growth and survey sample size as well as differences in survey response based on language preference among a Hispanic population. Percentages of item non-response on selected survey questions were compared for Hispanic respondents choosing to complete the survey in Spanish and those choosing to complete the survey in English. The mean number of attempts to complete the survey was also compared based on language preference among Hispanic respondents.

**Results:**

The sample size of Hispanic persons in the Behavioral Risk Factor Surveillance System saw little growth compared to the actual growth of the Hispanic population in the United States. Significant differences in survey item non-response for nine of 15 survey questions were seen based on language preference. Hispanic respondents choosing to complete the survey in Spanish had a significantly fewer number of call attempts for survey completion compared to their Hispanic counterparts choosing to communicate in English.

**Conclusions:**

Including additional measures of acculturation and increasing the sample size of Hispanic persons in a national health survey such as the Behavioral Risk Factor Surveillance System may result in more precise findings that could be used to better target prevention and health care needs for an ethnic minority population.

## Background

Recent US Census data indicate that the fastest-growing ethnic group in the United States is Hispanic, with a total estimated population of approximately 45 million persons in 2008 [1]. Projections by the US Census Bureau suggest that by the year 2050, the number of Hispanic persons in the US will more than double [2]. Because of the increase in the Hispanic population within the US, Spanish has become the primary language of many Hispanic households. In fact, in 2006, the US Census Bureau estimated that the number of persons aged 5 years and older who spoke predominantly Spanish and who reportedly spoke English "less than very well" was greater than 16 million [3].

The observed shift in the racial and ethnic demographics within the US has resulted in an increased body of research examining language preference among Hispanics in association with wide-ranging health outcomes such as health care delivery, utilization, and health behaviors. For example, researchers have reported that Spanish language preference is associated with barriers to access and use of health care services [4-6], receiving less-efficient care [7,8], and participation in risky health behaviors such as binge drinking [9]. Findings from these studies suggest that disparities in health care delivery exist due to a lack of Spanish literacy from the provider side and potential language barriers from the patient side. Therefore, non-English-speaking populations may be at a higher risk for developing preventable diseases because of the observed language barrier.

Based on the demonstrated differences in health outcomes for the Hispanic population compared to other ethnic populations, and within the Hispanic population based on language preference [4-9], there have been renewed efforts to study health disparities in this underserved group [10]. However, relatively few studies exist in the literature examining methodological issues in the surveying of health behaviors for Hispanic populations. Several of the documented issues include efficient sample sizes, issues of cooperation, and non-response based on cultural differences and language barriers, as well as development of Spanish language survey materials and training of survey staff [11].

The increase in disparities research among Hispanic persons in the US, combined with the paucity of published work on Hispanic survey methodology specifically related to differences based on language preference in this vulnerable population, presents a gap in survey knowledge that should be studied. Therefore, this study set out to describe recent trends and issues in the collection of language preference among Hispanic persons in the US, using a large, telephone health survey in states that offered a Spanish-language version of the survey.

## Methods

Data for this study were taken from five years (2003-2007) of the Behavioral Risk Factor Surveillance System (BRFSS) survey, [12] as well as from five years (2003-2007) of the US Census [13]. The BRFSS is a state-based, random-digit-dialed telephone survey collecting data on health risk behaviors, preventive health practices, and health care access primarily related to chronic disease and injury. Survey calls are made on weekdays as well as weeknights and weekends ranging from 10:00 A.M. to 9:00 P.M. Up to fifteen call attempts are made to complete each survey.

The five years of data from the BRFSS were used because they were the latest available BRFSS data and because Spanish-language surveys were first offered by states beginning in 2003. Between 29 and 32 states and US territories had surveys completed in Spanish for each of the five years. These states and territories included Arizona, Arkansas, California, Colorado, Connecticut, Florida, Illinois, Indiana, Kansas, Massachusetts, Missouri, Nebraska, Nevada, New Jersey, New Mexico, New York, North Carolina, North Dakota, Ohio, Rhode Island, South Carolina, Texas, Utah, Vermont, Virginia, Washington, Wyoming, Puerto Rico, and the US Virgin Islands. BRFSS questionnaires are translated into Spanish by an internal Centers for Disease Control panel of Spanish speakers. These surveys are then sent to states where regional dialects are incorporated. Analysis of response rates and item non-response in the BRFSS were limited to persons identifying themselves as Hispanic. Median cooperation rates for the BRFSS survey by year were as follows: 2003, 74.8%; 2004, 74.3%; 2005, 75.1%; 2006, 74.5%; and 2007, 72.1% [14].

The first analysis entailed comparing published US Census estimates for numbers of Hispanic, African-American, and white persons 18 years of age and older to the number of BRFSS surveys completed by Hispanic, African-American, and white persons 18 years of age and older. For our study, we also looked at the number of surveys completed in Spanish by Hispanic persons. Five years of data from the BRFSS and the census were compared for trends to gain a better perspective of sampling representation in the survey.

Three survey outcomes from the BRFSS were studied. First, trends in the numbers of Spanish language surveys were determined over a five-year period. These were compared both to trends in the numbers of surveys completed by various demographic groups and to demographic trends in the US. Second, differences in item non-response using a series of questions on health care access and health behaviors were examined based on language preference in an adult Hispanic population. Third, the number of attempts to complete a health telephone survey was examined based on language preference in the same adult Hispanic population.

Fifteen questions were selected from the core survey to be included in the analyses of non-response. These included three questions each from the following five categories: demographics, health services access and use, self-rated health status, chronic conditions, and health behaviors. Responses of "don't know/not sure" and "refused" to these questions were combined and considered to be non-responses for purposes of analysis.

For demographics, the three questions included marital status, educational attainment, and employment status. Marital status was determined with the question: "Are you married, divorced, widowed, separated, never married, or refused?" Educational attainment was determined with the question: "What is the highest grade or year of school you completed?" The choices included: "Never attended school or only attended kindergarten," "grades 1 through 8," "grades 9 through 11 (some high school)," "grade 12 or GED (high school graduate)," "college 1 year to 3 years (some college or technical school)," "college 4 years or more (college graduate), or refused." Employment status was determined by asking: "Are you currently employed for wages, self-employed, out of work for more than one year, out of work for less than one year, a homemaker, a student, retired, or refused?"

For health services access and use, the three questions included access to any health plan, multiple health care providers, and cost as a barrier to care. The first question was: "Do you have any kind of health care coverage, including health insurance, prepaid plans such as HMOs, or government plans such as Medicare?" Responses to this question could be "yes," "no," "don't know/not sure," or "refused." The second question was: "Do you have one person you think of as your personal doctor or health care provider?" Responses to this question could be "yes, only one," "more than one," "no," "don't know/not sure," or "refused." The third question was: "Was there a time in the past 12 months when you needed to see a doctor but could not because of cost?" Answers to this question could be "yes," "no," "don't know/not sure," or "refused."

Three questions on self-rated health status inquired about general health status, number of days that physical health was not good, and number of days that mental health was not good. General health status was determined with the question: "Would you say that your general health is excellent, very good, good, fair, poor, don't know/not sure, or refused?" Physical health status was determined with the question: "Now thinking about your physical health, which includes physical illness and injury, for how many days during the past 30 days was your physical health not good?" Respondents could list up to 30 days, or they could respond with "don't know/not sure," or "refused." Mental health status was determined with the question: "Now thinking about your mental health, which includes stress, depression, and emotional problems, for how many days during the past 30 days was your mental health not good?" Respondents could list up to 30 days or could provide a response of "don't know/not sure," or "refused."

Determination of the presence of three chronic conditions was included. These conditions were diabetes, hypertension, and asthma. The presence of diabetes was determined with the question: "Have you ever been told by a doctor that you have diabetes?" If the respondent was female and answered "yes," she was asked, "Was this only when you were pregnant?" Possible responses were "yes," "yes, but female told only during pregnancy," "no," "don't know/not sure," or "refused." The presence of hypertension was determined with the question: "Have you ever been told by a doctor, nurse, or other health professional that you have high blood pressure?" If the respondent was female and answered "yes," a follow-up question was asked: "Was this only when you were pregnant?" Possible responses were "yes," "yes, but female only told during pregnancy," "no," "don't know/not sure," or "refused." The presence of asthma was determined with the question: "Have you ever been told by a doctor, nurse, or other health professional that you have asthma?" The responses could be "yes," "no," "don't know/not sure," or "refused."

We also examined the outcomes of three health behaviors including exercise, ever having blood cholesterol checked, and ever having smoked. Participation in exercise activities was determined with the question: "During the past month, other than your regular job, did you participate in any physical activities or exercises such as running, calisthenics, gardening, or walking for exercise?" Responses to this question included "yes," "no," "don't know/not sure," and "refused." Cholesterol awareness was determined with the following: "Blood cholesterol is a fatty substance found in the blood. Have you ever had your blood cholesterol checked?" Responses to this question included "yes," "no," "don't know/not sure," and "refused." Ever having smoked was determined with the question: "Have you smoked at least 100 cigarettes in your entire life?" Responses to this question included "yes," "no," "don't know/not sure," and "refused."

### Statistical Analyses

All analyses of data using the BRFSS were conducted in SUDAAN [15] to account for the complex sampling design of the survey. We recorded the number of surveys completed each year by self-identified Hispanic persons, the number of surveys completed in Spanish by self-identified Hispanic persons, and the number of surveys completed by African Americans and whites. We compared these to US Census estimates for the Hispanic, African-American, and white populations 18 years of age and older, by year. Rates of change in percentages were calculated for each group over the five-year period.

Non-responses for questions in the five categories were stratified by language preference among self-identified Hispanics completing the survey. Differences in non-response rates, which are an indication of item appropriateness as well as survey completeness, between the two language groups were tested using Chi-square analyses for each question.

The number of call attempts to complete a survey were examined in two ways. The first was a comparison of the mean number of calls for each language preference. The difference in means was confirmed using a t-test. The second examination method utilized a linear regression model that looked at the influence of language preference on the number of call attempts while controlling for age and sex.

## Results

Over the period from 2003 to 2007, the number of Spanish-language BRFSS surveys completed by self-identified Hispanic persons increased by nearly 50%. From 2003 to 2007, the US Census estimates for the US Hispanic population increased by approximately 14%, and the total number of self-identified Hispanic persons completing the BRFSS survey increased by 59.3%. During the same time period, the US Census estimates for the African-American population increased by 6.7%, and the total number of African Americans responding to the BRFSS survey increased by 60.6%. The increase in the Hispanic population in the US from 2003 to 2007 was nearly seven times greater than that of African Americans. However, the numbers of BRFSS surveys completed by Hispanic persons was more than four times less than that of surveys completed by African Americans (Table 1).

**Table 1:** Number of Behavioral Risk Factor Surveillance System (BRFSS) surveys by Hispanic ethnicity and language preference and US Census population estimates for Hispanics, African Americans, and whites by year, 2003-2007

	2003	2004	2005	2006	2007
Number of self-identified Hispanic persons completing BRFSS Spanish language surveys	8,466	9,684	11,471	11,793	12,811
Number of self-identified Hispanic persons in BRFSS	19,652	23,015	25,539	26,432	31,310
Number of self-identified African-American only, non-Hispanic persons in BRFSS	20,684	26,031	27,735	28,772	33,216
Total number of BRFSS surveys	264,684	303,822	356,112	355,710	430,912
US Census estimates for US Hispanic population, 18 years of age and older	26,103,356	27,003,064	27,928,716	28,883,199	29,842,175
US Census estimates for US African-American population, 18 years of age and older	25,663,123	26,074,862	26,475,326	26,907,754	27,361,091
US Census estimates for US white population, 18 years of age and older	177,949,002	179,612,269	181,244,814	182,966,991	184,745,318
US Census estimate for total US population	290,447,644	293,191,511	295,895,897	298,754,819	301,621,157

Source: Behavioral Risk Factor Surveillance System and US Census Bureau

Among Hispanic respondents to the BRFSS, little difference was noted in age and sex between those choosing to complete the survey in English and those choosing to complete the survey in Spanish. The average age of English respondents was 38.9 years compared to 39.6 years for Spanish respondents. Among English respondents, 50.2% of the sample was male, compared to 49.6% among Spanish respondents.

When examining non-response by language preference, numerous significant differences were found. Hispanic persons choosing to complete the survey in English had a greater proportion of non-responses for five of the 15 questions. These questions included marital status, employment status, cost as a barrier to care, exercise, and having their cholesterol checked. All of these differences were significant at a *P *< 0.01. In comparison, Hispanic persons choosing to complete the survey in Spanish had a greater proportion of non-responses to four of the 15 questions, three of which were related to self-reported health status. These questions included general health, days of physical health not good, days of mental health not good, and diagnosis of diabetes. All of these differences were significant at *P *< 0.01 (Table 2).

**Table 2:** Percentage non-response* to select health survey questions among Hispanic respondents by language preference in the Behavioral Risk Factor Surveillance System, 2003 - 2007.

	Spanish	English	p - value**
	n = 54,225	n = 68,877	
**Demographics**			
Marital status	0.2%	0.5%	<.01
Education	0.6%	0.8%	0.1
Employment	0.6%	1.1%	<.01
			
**Health Services**			
Access to any health plan	0.5%	0.5%	0.8
Multiple health care providers	0.4%	0.4%	0.5
Cost as a barrier to care	0.2%	0.3%	<.01
			
**Health Status**			
General Health	0.5%	0.3%	<.01
Days physical health not good	1.9%	1.6%	<.01
Days mental health not good	2.0%	1.5%	<.01
			
**Chronic Conditions**			
Diabetes	0.2%	0.1%	<.01
Hypertension	0.1%	0%	0.2
Asthma	0.2%	0.2%	0.7
			
**Health Behaviors**			
Exercise	0.1%	0.7%	<.01
Cholesterol checked	0.8%	1.2%	<.01
Ever smoked	0.4%	0.4%	0.5

Source: Behavioral Risk Factor Surveillance System

* Non-response includes "not sure/don't know" and refused.

** Chi-square test

Significant differences were also found when examining the number of call attempts for completion of a survey. Hispanic respondents choosing to complete the survey in Spanish required an average of 6.8 attempts whereas Hispanic persons completing the survey in English required an average of 7.2 attempts. Using a T-test, this difference was significant at *P *< 0.01. Linear regression modeling showed that after adjusting for age and sex, the difference between Hispanic persons choosing Spanish to complete the survey and those choosing English to complete the survey was still significant at *P *< 0.01 (Table 3).

**Table 3:** Analysis of call attempts for survey completion among Hispanic adults in the Behavioral Risk Factor Surveillance System (BRFSS), 2003-2007.

3A. T-test comparing average number of call attempts for survey completion by language preference		
	**mean number of attempts, μ**	**P < .01**
Spanish, n = 54,225	6.8	
English, n = 68,877	7.2	

**3B. Linear regression comparing effects of language preference on number of call attempts for survey completion, controlling for age and sex**.		

	**β**	**P < .01**
Spanish, n = 54,225	-0.35	
English, n = 68,877	0.00	

Source: Behavioral Risk Factor Surveillance System

## Discussion

These are the first analyses to specifically examine the world's largest health telephone survey for differences in survey metrics for the Hispanic population in the United States. The initial analysis in this study illustrates both the scope and speed of changing Hispanic demographics in the US and how those changing demographics relate to a national health survey. This analysis further describes the trend in the numbers of Spanish-language surveys in the BRFSS. The use of a Spanish-language questionnaire for this survey may have contributed to an increase in the amount of health care data collected, which is necessary to study disparate outcomes for the Hispanic population in the United States, but was not specifically demonstrated in these analyses. Subsequent analyses demonstrated that differences in survey metrics were found between the two language preference groups in the Hispanic population. This finding points to opportunities for further survey research on the best methods for data collection in the Hispanic population.

We believe that the differences in item non-response and call attempts for survey completion found in this study might be explained by cultural differences between respondents choosing to complete the survey in Spanish and those choosing to complete the survey in English. The questions that were less likely to be answered by Spanish-speaking respondents included general health, days of physical health not good, days of mental health not good, and diagnosis of diabetes. Three of these questions deal with self-perceived health status, and it is possible that cultural beliefs within the Hispanic population, especially among those who are less acculturated (Spanish speaking), would affect responses to questions about self-perceptions of health. However, our finding that Hispanic respondents choosing to complete the survey in Spanish had a lower mean number of call attempts for survey completion was surprising. This difference may also be explained by further measures of acculturation, such as length of time in the country and country of origin, which are not included on the survey. Spanish-speaking respondents may choose to feel more associated with their host country by participating in a health survey, but may not fully understand some questions due to cultural context. These differences should be explored more fully, and findings from this study provide preliminary information for debate and research on the topic. These findings also speak to the necessity of including more measures of acculturation in the BRFSS in order to better understand health outcomes and survey metrics for a growing minority ethnic population.

Nearly 30 years ago, Lu Ann Aday and colleagues explored the evolution of different methodological issues in health surveys among the Hispanic population in the US [11]. These researchers described three generations of health services research beginning in the mid-1950s that evolved from participant observation to small non-probability studies of the Hispanic population to large probability-sampling surveys of health behaviors. Aday and her team commented that at that time there was little research on methodological issues of surveying Hispanic populations. This is still true today even though there are numerous studies using data from large health surveys to examine health outcomes among Hispanics based on differences in acculturation, including language preference. However, these studies are limited to few outcomes and even fewer predictive factors, due to limited data collection on behaviors and traits specific to the Hispanic/Latino community. Therefore, this current work brings attention to the need for increased data collection on Hispanic health issues and the need for more survey research in this field.

The estimates initially presented in our analyses demonstrate that the rate at which the Hispanic population has grown has outpaced that of the other traditional minority population in the US, African Americans, and demonstrates the increasing proportion of the US population made up of Hispanic persons. At the same time, the numbers of self-identified Hispanics completing the BRFSS have lagged behind those in the African-American community. These facts create an opportunity for a large health survey to focus on a growing and increasingly important demographic segment of our country. This opportunity could be addressed through increased federal funding for larger sample sizes and for oversampling of Hispanic populations throughout the country. Future surveys might include more in-depth questions examining cultural differences related to health care, and potentially increase response rates to surveys among newly immigrated and possibly less-acculturated populations.

A comprehensive literature review on acculturation and Latino health in the US by Lara and colleagues concluded that public health officials should promote the use of acculturation scales in all major government-sponsored health surveys and that these acculturation scales should include more than one or two measures [16]. The findings from our work have shown that language preference, one measure of acculturation, affects survey outcomes such as item non-response and number of contact attempts for survey completion.

It is possible that further measures of acculturation such as length of time spent in the US and country of origin may also have an effect on survey outcomes. These findings have significant implications for surveying the health of an ethnic/minority population and should be studied in data that collect this information. These implications include focusing on culturally appropriate questionnaire development for ethnic populations and assuring representative samples for minority populations.

## Conclusions

Including additional measures of acculturation and increasing the sample size of Hispanic persons in the Behavioral Risk Factor Surveillance System would result in more precise and more representative findings that could be used to better target prevention and health care needs for this ethnic minority population. These changes in data collection would be beneficial for determining public health issues among the Hispanic/Latino community, a growing and increasingly important portion of the United States population.

## Competing interests

The authors declare that they have no competing interests.

## Authors' contributions

WSP and WSG contributed to the design and conceptualization of the study, the analysis and interpretation of the data and the writing of the manuscript. ESF and LSB contributed to the acquisition and interpretation of the data and the writing of the manuscript. All authors have approved of the final draft of the manuscript.

## Authors' informations

WSP is an epidemiologist in the Division of Adult and Community Health at the Centers for Disease Control and Prevention in Atlanta, GA.
